# Innovation through Wearable Sensors to Collect Real-Life Data among Pediatric Patients with Cardiometabolic Risk Factors

**DOI:** 10.1155/2014/328076

**Published:** 2014-01-06

**Authors:** Kestens Yan, Barnett Tracie, Mathieu Marie-Ève, Henderson Mélanie, Bigras Jean-Luc, Thierry Benoit, Maxime St-Onge, Lambert Marie

**Affiliations:** ^1^Université de Montréal Hospital Research Center, Centre de Recherche du CHUM (CRCHUM), Tour St-Antoine S02-340, 850 St-Denis, Montreal, QC, Canada H2X 0A9; ^2^Social and Preventive Medicine Department, Université de Montréal, Montreal, QC, Canada H3N 1X7; ^3^CHU Sainte-Justine Research Center, Montreal, QC, Canada H3T 1C5; ^4^Department of Exercise Science, Concordia University, Montreal, QC, Canada H4B 1R6; ^5^Department of Kinesiology, University of Montreal, Montreal, QC, Canada H3T 1J4; ^6^Division of Endocrinology, Department of Pediatrics, CHU Sainte-Justine and Université de Montréal, Montreal, QC, Canada H3T 1C5; ^7^Division of Cardiology, Department of Pediatrics, CHU Sainte-Justine and Université de Montréal, Montreal, QC, Canada H3T 1C5; ^8^Synemorphose Inc., Montreal, QC, Canada H4C 3H2; ^9^Division of Genetics, Department of Pediatrics, CHU Sainte-Justine and Université de Montréal, Montreal, QC, Canada H3T 1C5

## Abstract

*Background*. While increasing evidence links environments to health behavior, clinicians lack information about patients' physical activity levels and lifestyle environments. We present mobile health tools to collect and use spatio-behavioural lifestyle data for personalized physical activity plans in clinical settings. *Methods*. The Dyn@mo lifestyle intervention was developed at the Sainte-Justine University Hospital Center to promote physical activity and reduce sedentary time among children with cardiometabolic risk factors. Mobility, physical activity, and heart rate were measured in free-living environments during seven days. Algorithms processed data to generate spatio-behavioural indicators that fed a web-based interactive mapping application for personalised counseling. Proof of concept and tools are presented using data collected among the first 37 participants recruited in 2011. *Results*. Valid accelerometer data was available for 5.6 (SD = 1.62) days in average, heart rate data for 6.5 days, and GPS data was available for 6.1 (2.1) days. Spatio-behavioural indicators were shared between patients, parents, and practitioners to support counseling. *Conclusion*. Use of wearable sensors along with data treatment algorithms and visualisation tools allow to better measure and describe real-life environments, mobility, physical activity, and physiological responses. Increased specificity in lifestyle interventions opens new avenues for remote patient monitoring and intervention.

## 1. Introduction

The rising prevalence of obesity and cardiometabolic risk observed among youth has led to predictions of decreased life expectancy among the next generation of North Americans, a first in history [1]. The American Heart Association has reclassified obesity as a “major, modifiable” risk factor for coronary heart disease (CHD) and diabetes [2]. This condition is modifiable through dietary and physical activity changes [3].

Classical clinical interventions promoting a healthy lifestyle are based primarily on counseling not always tailored to individual's profile and on structured exercise programs that have proven to be complex, costly to maintain, and have long-term poor adherence. Sustainable interventions need to focus on interindividual specificity [4, 5] and the development of personalized activity plans [6]. Advances in mobile health and wearable devices offer new ways to collect and interpret data on environments, behaviours, physiology and well-being. Recently, a clinical cardiac rehabilitation intervention among adults using a wearable Electrocardiogram (EKG), a Global Positioning System (GPS) receiver, and a smartphone for real-time data transmission on exercise sessions [7] showed significant improvements in walking distance, depression, and the physical component of the SF36 general health questionnaire. To our knowledge, no clinical lifestyle intervention targeting children and youth has integrated the use of GPS, accelerometers, and heart rate monitors. This paper describes the use of multiple technologies to integrate real-life information in tailored clinical lifestyle interventions in youth. Proof of concept and feasibility is illustrated using baseline data collected among the 37 first participants of the Dyn@mo intervention.

## 2. Methods

### 2.1. The Dyn@mo Intervention

The Dyn@mo lifestyle intervention (Sainte-Justine University Hospital Center, Montreal, Canada) targets children and adolescents aged 6 to 17 years old with cardiometabolic risk factors, such as obesity, hypertension, disorders in glucose regulation, or dyslipidemia. Its primary goal is to promote physical activity and reduce sedentary time to improve children's cardiometabolic profile. To do so, the intervention relies on gathering data on mobility and physical activity using wearable sensors. These data provide a detailed picture of real-life conditions and physical activity levels, improving the health care professional's ability to tailor counseling. This paper presents these tools and baseline pilot data—that is, GPS and accelerometry collected for seven days after the first visit—among the first 37 patients who enrolled in the Dyn@mo program between March and November 2011.

The intervention comprises several clinical encounters and regular followup by phone and email. On three occasions (0, 12 months, and 24 months), children are equipped with a heart rate monitor, a GPS receiver, and an accelerometer to collect heart rate, daily mobility, and physical activity data during a 7-day period (results presented here use baseline, that is, data collected for seven days after first visit at 0 month). Four to six weeks later, patients, families, and professionals visualise resulting indicators which could be called a “spatio-behavioural diagnosis” using a map-based interactive web application. The next sections present the tools developed for such continuous monitoring and analysis of real-time data, including (i) the data collection tools and methods, (ii) the data treatment expert system, and (iii) the map-based interactive web application for rendering of spatio-behavioural information to patients and caregivers. Then, data obtained from the 37 first patients enrolled in the program are presented, along with some exploratory statistics linking spatial, behavioural, and environmental characteristics.

#### 2.1.1. Data Collection Tools and Methods

During an initial visit at the clinic, patients provide home and school addresses and report some of their regular destinations. This information is integrated in the spatio-behavioural web application. Patients are equipped with a GPS receiver, an accelerometer, and a heart rate monitor. They are instructed on device usage—wearing the accelerometer at the hip, the heart rate monitor on the chest during all waking hours, and carrying the GPS device with them at all times and charging it overnight. A return envelope is provided to send the devices back through regular mail after completion of the 7-day period. The monitoring hardware configuration includes a Trimble Juno SC GPS unit (http://www.trimble.com/), an ActiGraph ActiTrainer activity monitor (http://www.theactigraph.com/), and a Polar Wearlink Heart transmitter chest monitor with wireless link to the activity monitor receptor. The GPS device has a manufacturer-reported spatial accuracy of 1–3 meters and is configured to collect location information over an epoch of one second. Collected coordinates are saved in an ArcGIS shapefile (.shp) format by a coding procedure that was developed under ArcPad and installed on the Trimble device within the Windows Mobile 6 platform. Shapefiles are compressed and stored on local memory every hour. The ActiTrainer accelerometer is configured to record counts for each axis at a one minute epoch. The HR monitor records heart beats per minute. Data are saved on the local memories of the GPS device for GPS tracking and of the physical activity monitor for HR and accelerometry. Upon reception of the devices at the clinic, data files are uploaded on a desktop computer for further processing.

#### 2.1.2. Data Treatment Expert System

An expert panel composed of epidemiologists, kinesiologists, pediatricians, cardiologists, geomaticians, and geographers worked on defining relevant indicators to support the lifestyle promotion intervention. The indicators were classified as follows: (a) physical activity and sedentary behaviour indicators, (b) spatial indicators and (c) combined indicators of physical activity and spatial behaviour, and finally (d) usage/device performance indicators.

An ArcGIS ArcToolBox was developed using Python language to automatise data treatment, that is, to derive indicators from raw GPS, accelerometry, and heart rate data files and generate a synthetic XML output file for web integration. The XML file contains data to be visualised through the online application, including geographic information of activity locations [8], GPS tracks of trips, accelerometry-related information, and HR data. The procedure is automated—an analyst runs the ArcToolBox on the raw data, which generates the XML file. Further qualification of detected activity locations can be made through the web application.

Raw data files are validated and cleaned prior to indicator construction. Poor quality datapoints are eliminated and missing GPS data are imputed according to specific rules outlined below. Accelerometry periods of 60 consecutive minutes with zero counts or more are considered as nonvalid/nonwear time [9, 10]. Days with less than 10 hours of valid accelerometry measures are discarded [11]. GPS data points are cleaned according to measures of precision related to the configuration of the satellites (dilution of precision, DOP values) and speed (values with horizontal DOP > 8 or vertical DOP > 15 or positional DOP > 13 and speed > 130 km/h are removed). Given that GPS tracks are rarely continuous because of loss of signal due to nonvisibility of satellites, particularly when inside homes or buildings, missing GPS data points are imputed according to time and proximity rules. Data points are interpolated for gaps of 2 to 60 minutes. No interpolation is done if the gap is over 60 minutes, except if the two observed consecutive points are less than 100 m apart. Raw GPS tracks are processed to identify activity locations and related visits.

#### 2.1.3. Map-Based Interactive Web Application for Counseling

A web-based application supports data handling, data visualisation, and tailored counseling. Developed in a html and Flash programming environment, it runs on a IIS 7.0 web server with a Microsoft SQL Server 2008 database deployed on the hospital's internal servers. After login, a caregiver has access to his/her patient's files. An administration page provides information on patient's intervention in terms of upcoming, planned, or completed meetings, advancement of data collection and upload, state of activity profile, and counseling reports. A web form allows registration of basic information including home and school address. Maps of the home and school areas are automatically generated, to facilitate validation of locations by participants. A complementary mapping module documents relevant activity locations, such as actual places used for physical activity, or potential activity places, that is, sport centers, community centers, outdoor recreational areas, friend's homes, and so forth. The interactive map allows searching destinations through a textual search box connected to the Google Map API which will suggest results, or directly through the placement of a marker on the map. An infowindow allows collecting qualitative information for a documented location. This allows the mapping of “opportunities” for physical activity which can be enriched by contributions of the child, the family, and the caregiver.

**Figure 1:**
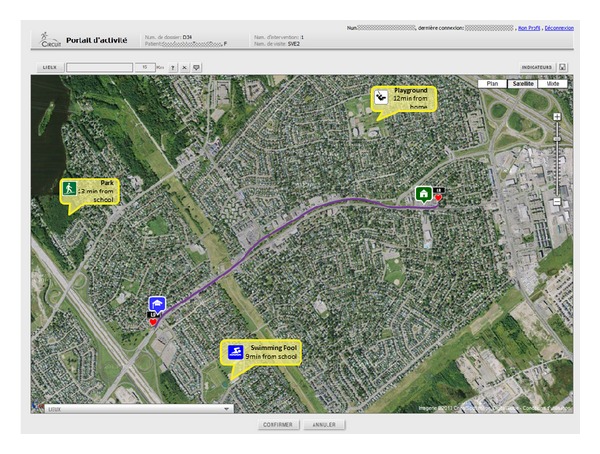
Interactive application for spatio-behavioural data visualization: patients' residential neighbourhood and mapping of opportunities.

**Figure 2:**
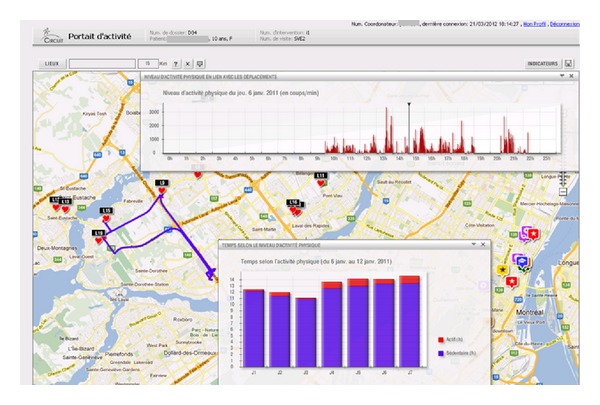
Interactive application for spatio-behavioural data visualization: GPS tracks, activity locations, and corresponding physical activity levels. NB: map indicates GPS tracks for one day automatically detected activity locations. Upper graph indicates physical activity levels during the day in relation to location (track). Lower graph shows time spent per physical activity level for each day.

Expert-system generated indicators of physical activity, spatial information or combinations thereof are visualised through an interactive module through tables, graphs, or maps (see example of visual presentation of patient's residential neighbourhood and opportunities in Figure 1 and additional graphs and maps in Figure 2). This visual interface is used by health care professionals to better understand the child's spatio-behavioural dynamics, and as a tool to communicate with the child and its family members and tailor physical activity counseling. All visual information such as maps or graphs as well as complementary text such as goals and objectives can be saved and reorganised at will in the evaluation report module. The report becomes part of the patient's file. It is saved on a secure server, printed and handed to the family.

### 2.2. Participants and Measurements

The discussed tools and methods are illustrated using baseline data from the 37 first participants aged 6 to 17 who were enrolled in the program between March and November 2011. All patients had a specific cardiovascular risk factors (e.g., diabetes, hypercholesterolemia, hypertension, obesity, etc.). Patients and parents signed an informed consent authorising data use for research purposes. The Dyn@mo intervention and its related research program were approved by the Sainte-Justine hospital ethics committee. Physical activity and spatio-behavioural indicators were compiled and compared between primary school-aged and secondary school-aged children. Residential neighbourhood variables were further compiled within a Geographic Information using a 500 meter network-buffer centered on place of residence. Variables of interest included 2006 Census-derived population density, proportion of immigrants, proportion of population with a university degree, household income, greenness using Landsat TM-5 satellite images and computing the Normalised Difference Vegetation Index (NDVI) [12], and street connectivity [13, 14], that is, the number of four-way intersections per square kilometer. All these variables have been associated with physical activity or walking in previous studies [15, 16].

## 3. Results

Summary statistics are provided in Table 1. Among the 37 initial participants, three had abnormally high accelerometry-derived step counts (above 55,000 steps a day in average) and were considered as outliers and removed from accelerometry analysis. Among the 34 remaining patients, 24 were females, and 10 males. Average standardised Body Mass Index (zBMI according to WHO growth curves) was 3.16 (SD = 0.711). BMI for age classified 33 out of 34 patients as obese (i.e., above the 95th percentile) and one patient as overweight (between the 85th and 95th percentile).

### 3.1. Device Usage/Performance

Days with at least 10 hours of valid accelerometry measures were retained. Three participants provided less than 4 valid days, 27 participants 5 days or more, 22 participants 6 days or more, and 11 participants provided valid accelerometry data for the full seven-day period. On average, a valid day included a total of 12 hours 54 minutes of wear time (SD = 59 minutes). Among the 34 participants, 33 had GPS data. Valid interpolated GPS data covered the full seven-day period for some 18 participants, 6 days for 3 participants, 5 days for 4 participants, and less than four days for three others. After data correction and imputation, cumulated GPS data was available on average for 17 hours and 10 minutes per day. GPS data was mostly imputed while being at home or at school (72%). Heart rate data was available for 34 participants. Only one participant provided less than 4 valid days, 31 participants 5 days or more, 27 participants 6 days or more, and 24 participants provided heart rate data for the full seven-day period.

### 3.2. Sensor Results

#### 3.2.1. Physical Activity

Accelerometry-derived data show 7,596 steps per day in average (SD = 2,315), with an average of 7,771 (2,497) steps recorded during weekdays and 6,609 (2,924) steps recorded during weekend days (nonsignificant difference, *P* = 0.577). Accelerometry count data revealed that participants spent an average of 10 hours 41 minutes in sedentary (<760 count per minute, CPM), one hour and 36 minutes in light (between 760 and 1951 CPM), and 36 minutes in moderate to vigorous physical activity (MVPA, above 1951 CPM). MVPA time during weekend days was significantly lower compared to week days (25 versus 38 minutes per day, *P* = 0.01).

#### 3.2.2. Heart Rate

Heart rate data showed some inconsistencies, particularly some very low counts (minimum heart rate beats of 20 beat per minute). The average recorded that heart rate was 80 beats per minute. Because of low observed values, reliability of these measures is questioned.

#### 3.2.3. Spatial Behavior

Thirteen participants lived within 1 km from their school, 15 within 1.6 km, 18 within 3 km, and 8 more than 10 km away. GPS data processing revealed that participants visited some 6.42 distinct activity locations on average and visited these locations in average 17.18 times. When establishing the convex hull polygon encompassing all GPS data points collected in a day—a measure of the area covered by a participant through his/her daily travelling—the median activity space area was 18.3 km^2^ for week days, and 1.7 km^2^ for weekend days. Some participants covered relative large areas with maximum values at 1,094 km^2^ and 316 km^2^, respectively. Participants also lived in areas with a range of density and socioeconomic profiles, as indicated by relatively large standard deviations in neighbourhood SES measures (See Table 1).

Exploratory statistics of bivariate associations between physical activity and spatial behaviour showed that the average number of steps was negatively associated with home-school distance during weekdays (−0.399, *P* = 0.020), but not during week-end days (−0.117, *P* = 0.553). Yet, when separating primary school aged children (11 and younger) from high-school aged children (12 and up), correlations became nonsignificant within each group, possibly due to a too small sample.  Table 2 shows significant differences in spatio-behavioural measures between these two groups. High-schoolers attend schools further away from home, cumulate less steps daily both during week days and weekend days, have shorter times of MVPA, and have larger activity spaces than their younger counterparts.

## 4. Discussion

This paper presents a series of tools supporting the Dyn@mo lifestyle intervention of the Sainte-Justine University Hospital Center, which targets lifestyle changes among children and youth with cardiometabolic risk factors. These tools include an objective seven-day evaluation of children's daily mobility and physical activity using three wearable sensors: a GPS receiver, an accelerometer, and a heart rate monitor. A semiautomated algorithm processes collected data to derive relevant indicators on patients' health behaviour, mobility, and life geographies. A web-based application further allows handling, visualization, and sharing of data between patients and their health practitioners. These novel tools and corresponding indicator statistics facilitate the integration of objective behavioural and physiological measures, as well as the patient's environmental constraints and opportunities, for a personalized, tailored lifestyle intervention.

With high levels of compliance and good data coverage over a 7-day period, this study demonstrated that wearable devices could be used in a clinical lifestyle intervention to collect real-life data among children. Furthermore, data processing algorithms allowed indicator construction and data restitution through an interactive mapping and graph-enabled web application. These tools and data provide information on patients' day-to-day environmental constraints and opportunities and can thereby contribute to lifestyle counseling.

More precisely, the use of a GPS device proved useful to document spatial behaviour and reveal the types of urban environments participants were exposed to in their everyday geographies. Combined to accelerometry data, it further allowed to *situate* physical activity, that is, understand when and where health behaviours such as sedentary behaviour or MVPA were occurring. The mapping of activity locations and trips, along with an interactive mapping capacity for identification of resources, further provided ways to visualize accessibility to opportunities, such as sports clubs or parks or other physical activity installations.

### 4.1. Limitations

A series of limitations apply to the tools and methods used to support the Dyn@mo lifestyle intervention. The analysis and interpretation of accelerometry data to evaluate active living and physical activity require further refinement. We used cut-off points recommended by the Actilife 4.3 User's Manual [17], yet, the distinction between nonwear time and sedentary time, the derivation of step counts, or transformation of accelerometry counts into levels of physical activity or energy expenditure estimates—that is, in part, establishing valid age- and BMI-specific cut-off points—remain contentious issues in pediatrics. Recent proposals have been made for nonwear/sedentary wearing time and could be tested [18], and comparative analyses have assessed the performance of different cut-off points and predictive equations of energy expenditure among children and youth [19]. However, although pediatric obesity is on the rise and a major concern, relatively few accelerometry validation studies have been done among obese children [20–22]. Concerning the use of a GPS device, issues related to battery life, manipulation errors, or device limitation such as nonfix or imprecision in measurement, are well described [23, 24]. However, statistics showed good wearing times and satisfying collection of GPS tracks. Another limitation pertains to the use of the chest-mounted heart rate monitor. Data analysis clearly revealed measurement errors, with abnormal values—nonlife supporting—beat per minutes recorded among some participants. Adequate wearing of such devices may be problematic for longer periods and in free-living environments. For example, it is recommended to wet the electrode area of the chest belt for proper functioning. This may generate discomfort or electrode contact issues which may reduce compliance or bias the readings towards lower numbers. Alternatively, ways of providing instructions on wearing and handling procedure may need to be improved.

Future of wearable sensors for clinical interventions: use of GPS for understanding spatial behaviour and exposure to environments is relatively new but rapidly gaining momentum [25]. Pilot and feasibility studies have demonstrated the capacity of GPS to locate health behaviours, with a potential to better understand environmental influences [26]. For example, GPS units have been used to track travel patterns among adolescents [27, 28], analyze walking among adults [29], analyze bicycling routes in relation to existing road infrastructures [30], mobility patterns among older adults [31, 32], link mobility with mental health outcomes [33, 34], analyze active transportation [35–37] or relations between PA, and the built environment [38, 39]. GPS data have also been used to validate parent-reported questionnaires on children's activity locations [40], with results showing significant place misclassifications in parent-reported activity locations and times, thus underscoring the usefulness of GPS systems for obtaining reliable information on activities and locations. Along that line, novel map-based questionnaires also allow the collection of regular destinations for improved exposure assessment and may be used in place of or in complement to GPS tracking [41].

Yet, use of GPS devices to support clinical lifestyle interventions is still rare. Recently, a clinical cardiac rehabilitation intervention used a wearable Electrocardiogram (EKG), a GPS receiver, and a smartphone for real-time data transmission to monitor walking-based exercise sessions in real-time [7]. Participants showed statistically significant improvements in walking distance, depression, and the physical component of the SF36 general health questionnaire. Use of wearable sensors was however limited to short periods—exercise times—and on adult patients only.

Further research will need to explore how GPS- and complementary sensor based spatio-behavioural indicators such as those proposed here are associated to cardiometabolic profiles. This would reinforce the validity of using such lifestyle indicators for intervention. Measures of accessibility to food sources or opportunities for physical activity in proximity to regular destinations could further serve to analyse actual health behaviour and cardiometabolic profile [42].

### 4.2. Data Capture, Data Processing, and Applications

Challenges in mobile health arise along the three phases of what can be seen as a three-tier continuum going from data capture to data processing to data usage.

Beyond measurement validity issues, challenges in data capture include practical considerations such as relatively short continuous sensing time linked to poor battery capacities, particularly for GPS; difficulty of manipulation; a relatively poor integration of sensors generally implying to wear several distinct devices; and a relative lack of integrated communication protocols which limits linkage between sensors or between sensors and cellphone networks, which limits real-time tracking and feedback to the user. In order to address some of these issues, team members have developed a novel integrated multisensor device containing a GPS module, a triaxial accelerometer, and two means of communication: a GPRS module for cellphone network data transmission and an ANT+ module for local 2.4 GHz data transmission, which allows addition of external sensors such as accelerometers or continuous glucose monitors, or home-based sensors such as RFID tags. Real-time data transmission capacities allow distant patient monitoring, or feedback through web or mobile applications. Whereas increasing ubiquity of smartphones—which provide a series of embedded sensors—may represent an important potential in the future [43, 44]. Yet, various issues including relatively poor battery life or unknown validity of embedded sensors in addition to continuous updates of new hardware/software configurations clearly limit current applicability in clinical settings.

Data processing is key because continuous monitoring generates large amounts of raw data, which need to be transformed and synthesized to be useful, both for the patient and the clinician. For example, there is a need for clear documentation of how raw GPS data is transformed into spatio-temporal indicators, how raw accelerometry data is transformed into energy estimates. Physical activity estimates should not be based on undocumented procedures providing proprietary “counts” of physical activity. More research—and documentation—in the creation of useful spatial/behavioural/physiological indicators is needed and will require multidisciplinary perspectives including contributions from nonhealth fields such as geography, computer science, or engineering. To support increasing real-time data streams coming from a variety of sensors, novel information system architectures also need to be developed. Sound and secure cloud architectures will help move toward big real-time data which in turn will then support the development of advanced machine learning algorithms for improved performance.

Among the applications of such systems, feedback loops to the user cannot only improve health management but also further data collection—for example, using web or mobile based prompted recall applications [45–47].

Further research should evaluate patients' and physicians' perception and usage of such tools and methods and better assess how sensor-based information is best put at use. Caution is required, because although the potential is promising, both across age groups and across health domains, unintended harmful consequences can arise. Yet, because such development require large ranges of expertise, research funding schemes also need to be able to support interdisciplinary teams reaching beyond the sole domain of population health or clinical research, to computer science and engineering.

## Figures and Tables

**Table 1:** Summary baseline statistics of Dyn@mo participant (*n* = 34).

Variable	*N*	Min	Max	Average	Std dev.
Individual profile					
Age	34	6	17	11.1	3.1
BMI	34	21.7	60	32.2	7.3
BMI for age/gender percentile	34	94.8	99.9	98.59	1.26
zBMI	30	1.96	4.79	3.16	0.71
Neighbourhood characteristics					
Population density (km^2^)	34	0.9	74735	7330	13551
Immigrants (%)	34	0	71	18	21
With university degree (%)	34	0	55	19	13
Household income ($)	34	35.125	140.495	73.983	25.480
Greenness (mean NDVI)	34	−0.25	0.239	0.006	0.132
Street connectivity (4+ way intersections) (km²)	34	0	85.05	19.89	23.19
Home-school road network distance (m)	34	139	24.428	5.800	6.697
Device usage					
Nb of valid accelerometer days (>10 h)	34	1	8	5.59	1.62
Average time with accel. data per valid day	34	10:35	15:08	12:54	0:59
Nb of days with heart rate data	34	3	8	6.47	1.19
Nb of days with GPS data	33	1	11	6.1	2.1
Daily average GPS time/recorded (hh:mm)	33	01:09	19:04	11:47	04:55
Daily average GPS time/corrected (hh:mm)	33	07:01	23:54	17:10	04:55
Daily average missing GPS time (hh:mm)	33	00:05	16:58	06:49	04:09
Physical activity					
Average number of steps per day					
All days	34	3.060	12.344	7.596	2.315
Weekdays	34	2.334	13.488	7.771	2.497
Weekend days	28	1.219	15.900	6.609	2.924
Time sedentary (hh:mm)					
All days	34	07:27	12:35	10:41	01:08
Weekdays	34	07:27	13:23	10:49	01:14
Weekend days	28	06:21	12:36	10:19	01:25
Time in light activity (>760 and <1951 counts/min)					
All days	34	00:35	02:39	01:36	00:33
Weekdays	34	00:27	02:41	01:35	00:35
Weekend days	28	00:21	02:49	01:36	00:37
Time in moderate to vigorous activity (>1951 counts/min)					
All days	34	00:07	01:17	00:36	00:19
Weekdays	34	00:07	01:22	00:38	00:20
Weekend days	28	00:00	01:46	00:25	00:19
Number of days with >30 min of moderate to vigorous activity	34	0	7	2.91	2.08
Heart rate (beats per minute)					
All days	34	20	104	80.8	21.8
Weekdays	34	24	106	81.8	21.7
Weekend days	28	0	110	74.5	30.3
Spatial behaviour					
GPS: weekly average of number of activity locations	33	1	21	6.42	5.16
GPS: weekly average of visits	33	1	52	17.18	12.095
Activity space size (km²)					
All days	33	.0	1.094	25.69 (median)	246.1
Weekdays	32	.0	1.036	18.28 (median)	232.9
Weekend days	29	.0	316	1.69 (median)	67.7

**Table 2:** 

	Primary school children		Secondary school children		*t*	Ind. samples *t*-test sig.
	Average (age < 12)	*n*	Average (age ≥ 12)	*n*		
Home-school distance (meters)	2.491	19	9.991	15	−3.615	0.002
Accel: average steps per valid day (weekdays)	8.983	19	6.237	15	3.507	0.002
Accel: average steps per valid day (weekends)	7.997	15	5.008	13	3.174	0.004
Accel: moderate to vigorous PA time (weekdays)	00:45	19	00:29	15	2.356	0.026
Accel: moderate to vigorous PA time (weekends)	00:30	15	00:19	13	1.489	0.152
Activity space size (km^2^) (GPS) (weekdays)	26.5	17	169.5	15	−1.687	0.113
activity space size (km^2^) (GPS) (weekends)	26.3	16	50.8	13	−0.906	0.378
